# Novel Hydraulic Vulnerability Proxies for a Boreal Conifer Species Reveal That Opportunists May Have Lower Survival Prospects under Extreme Climatic Events

**DOI:** 10.3389/fpls.2016.00831

**Published:** 2016-06-09

**Authors:** Sabine Rosner, Jan Světlík, Kjell Andreassen, Isabella Børja, Lise Dalsgaard, Robert Evans, Saskia Luss, Ole E. Tveito, Svein Solberg

**Affiliations:** ^1^Institute of Botany, BOKU ViennaVienna, Austria; ^2^Centre MendelGlobe – Global Climate Change and Managed Ecosystems, Mendel UniversityBrno, Czech Republic; ^3^Norwegian Institute of Bioeconomy ResearchÅs, Norway; ^4^CSIRO Materials Science and EngineeringClayton, VIC, Australia; ^5^Norwegian Meteorological InstituteOslo, Norway

**Keywords:** climatic extremes, conduit wall reinforcement, functional wood anatomy, global warming, Norway spruce, *Picea abies*, top dieback

## Abstract

Top dieback in 40–60 years old forest stands of Norway spruce [*Picea abies* (L.) Karst.] in southern Norway is supposed to be associated with climatic extremes. Our intention was to learn more about the processes related to top dieback and in particular about the plasticity of possible predisposing factors. We aimed at (i) developing proxies for *P*_50_ based on anatomical data assessed by SilviScan technology and (ii) testing these proxies for their plasticity regarding climate, in order to (iii) analyze annual variations of hydraulic proxies of healthy looking trees and trees with top dieback upon their impact on tree survival. At two sites we selected 10 tree pairs, i.e., one healthy looking tree and one tree with visual signs of dieback such as dry tops, needle shortening and needle yellowing (*n* = 40 trees). Vulnerability to cavitation (*P*_50_) of the main trunk was assessed in a selected sample set (*n* = 19) and we thereafter applied SilviScan technology to measure cell dimensions (lumen (*b*) and cell wall thickness (*t*)) in these specimen and in all 40 trees in tree rings formed between 1990 and 2010. In a first analysis step, we searched for anatomical proxies for *P*_50_. The set of potential proxies included hydraulic lumen diameters and wall reinforcement parameters based on mean, radial, and tangential tracheid diameters. The conduit wall reinforcement based on tangential hydraulic lumen diameters ((*t*/*b*_ht_)^2^) was the best estimate for *P*_50_. It was thus possible to relate climatic extremes to the potential vulnerability of single annual rings. Trees with top dieback had significantly lower (*t*/*b*_ht_)^2^ and wider tangential (hydraulic) lumen diameters some years before a period of water deficit (2005–2006). Radial (hydraulic) lumen diameters showed however no significant differences between both tree groups. (*t*/*b*_ht_)^2^ was influenced by annual climate variability; strongest correlations were found with precipitation in September of the previous growing season: high precipitation in previous September resulted in more vulnerable annual rings in the next season. The results are discussed with respect to an “opportunistic behavior” and genetic predisposition to drought sensitivity.

## Introduction

Managed and unmanaged boreal conifer forests provide ecosystem services, such as climate regulation including carbon fixation (Gauthier et al., 2015; McDowell et al., 2016), and their role in national and rural economy of the Nordic countries is fundamental (Schlyter et al., 2006). So far, conifer forests of the northern hemisphere reacted to warming with an acceleration in growth (Kauppi et al., 2016) and some might have retained resilience to cope with current disturbances, but projected climate change scenarios (IPCC, 2013) suggest a threat to their health (Gauthier et al., 2015; McDowell et al., 2016). A positive effect of a warmer climate is that the period of net carbon uptake will be extended in the autumn, which could increase total carbon uptake in boreal forests dominated, for instance, by Norway spruce [*Picea abies* (L.) Karst.; Stinziano et al., 2015]. However, it is questionable if Norway spruce, an autochthonous species of the alpine timberline (Mayr et al., 2003, 2014) and of northern regions (Solberg, 2004; Andreassen et al., 2006), can cope with extreme weather events such as frequent and prolonged summer droughts (Schlyter et al., 2006). In that respect, lower latitude northern Norway spruce forests are more endangered as increase in mortality due to summer droughts has already been reported for southern Norway (Solberg, 2004; Hentschel et al., 2014). In this study, we focus on hydraulic vulnerability traits of healthy looking and declining Norway spruce trees and on the correlation of these traits to climate (extremes). We take advantage of an existing hydraulic dataset (Rosner et al., 2016) and define novel anatomical functional traits for trunkwood based on SilviScan technology (Evans, 1994, 1999).

Many conifer species have quite high hydraulic safety margins; their *P*_50_, i.e., the water potential resulting in 50% conductivity loss, is much lower than minimum water potentials measured out in the field (Choat et al., 2012). Compared to angiosperms, conifers are supposed to have a lower capacity to reverse embolism, thus to refill conduits and restore a hydraulically functional state (McDowell et al., 2008; McDowell, 2011; Meinzer and McCulloh, 2013; Zwieniecki and Secchi, 2015). Norway spruce growing at the timberline has the capacity to refill embolism induced in winter by freezing (Mayr et al., 2014). Moreover, Norway spruce has to some extent the ability to adapt the structure of wood to function optimally in local conditions (Gričar et al., 2015). Could this plasticity be disadvantageous under the impact of a sudden drought? Currently, there is no knowledge on the relationship among climate and anatomical traits associated to hydraulic vulnerability in Norway spruce. A precondition to establish this knowledge is the development of hydraulic predictive traits based on experimentally assessed reference data for *P*_50_ (Dalla-Salda et al., 2011). Recent studies showed that e.g., radial tracheid dimensions are influenced by climate (Castagneri et al., 2015; Gričar et al., 2015) but we lack proof if these traits are related to hydraulic vulnerability. Moreover, in many cases, trees that did not survive extreme climatic events are not available for analysis, since they were harvested in order to avoid e.g., bark beetle outbreaks. Therefore, study sites where healthy trees are compared to declining or dead trees are helpful to learn more about functional anatomical preconditions triggering tree mortality after extreme climate events (e.g., Britez et al., 2014; Hentschel et al., 2014).

For our study we selected two sites in SE Norway, where during the last 20 years an unusual symptom of top-dieback was observed on Norway spruce [*Picea abies* (L.) Karst.; Solberg, 2004]. The afflicted trees were usually 40–60 years old, dominant or co-dominant in the vigorously growing stands, often on former agricultural lands. First visible symptoms appeared as a stunted top growth, followed by needle discoloration and finally drying of the top crown. The trees died within 1–4 years from the onset of the symptoms. Trees with top-dieback were typically found scattered throughout the stand. So far, we know that decrease in sap flux density can occur during a quite short period (Børja et al., 2016), trees prone to top dieback had a less strict stomatal control and produced wood with lower density prior and during a period of drought stress compared to healthy looking trees (Hentschel et al., 2014; Rosner et al., 2014). Wood density is not directly causally linked to hydraulic vulnerability (*P*_50_); it is a proxy where statistical correlations exist because some traits that have an effect on density also affect hydraulic properties (Hacke et al., 2001; Bouche et al., 2014; Lachenbruch and McCulloh, 2014). Once such indirect structure-function relationships are established, their application as screening tools is justified because density and other anatomical parameters are relatively easy to assay, whereas the underlying direct hydraulic measurements are labor intensive and prone to errors (Cochard et al., 2013). However, when taking advantage of proxies such as wood density it should be clear that they are only valid over the conditions used (Lachenbruch and McCulloh, 2014) and thus neither applicable for other species nor for other stem parts (branches) or organs (roots). Another advantage of anatomical proxies for hydraulic properties is that their assessment is independent on the current functional state of secondary xylem (i.e., wood) at the time of harvesting; other than direct hydraulic measurements, proxies allow us to make assumptions on the hydraulic vulnerability of wood that was produced many years ago and that may even not conduct sap vertically anymore (Rosner et al., 2014).

Aims of our study were, (i) to develop hydraulic vulnerability proxies for *P*_50_ based on anatomical data assessed on Norway spruce trunkwood by SilviScan technology and (ii) to test these proxies for their plasticity regarding climate, in order to (iii) analyze annual variations of hydraulic proxies of healthy looking trees and trees with top dieback and hence their impact on tree survival. Our intention was to learn more about the processes related to top dieback in Norway spruce and in particular about the plasticity of possible predisposing factors.

## Materials and methods

### Study sites, plant material, and sampling

**Figure 1:**
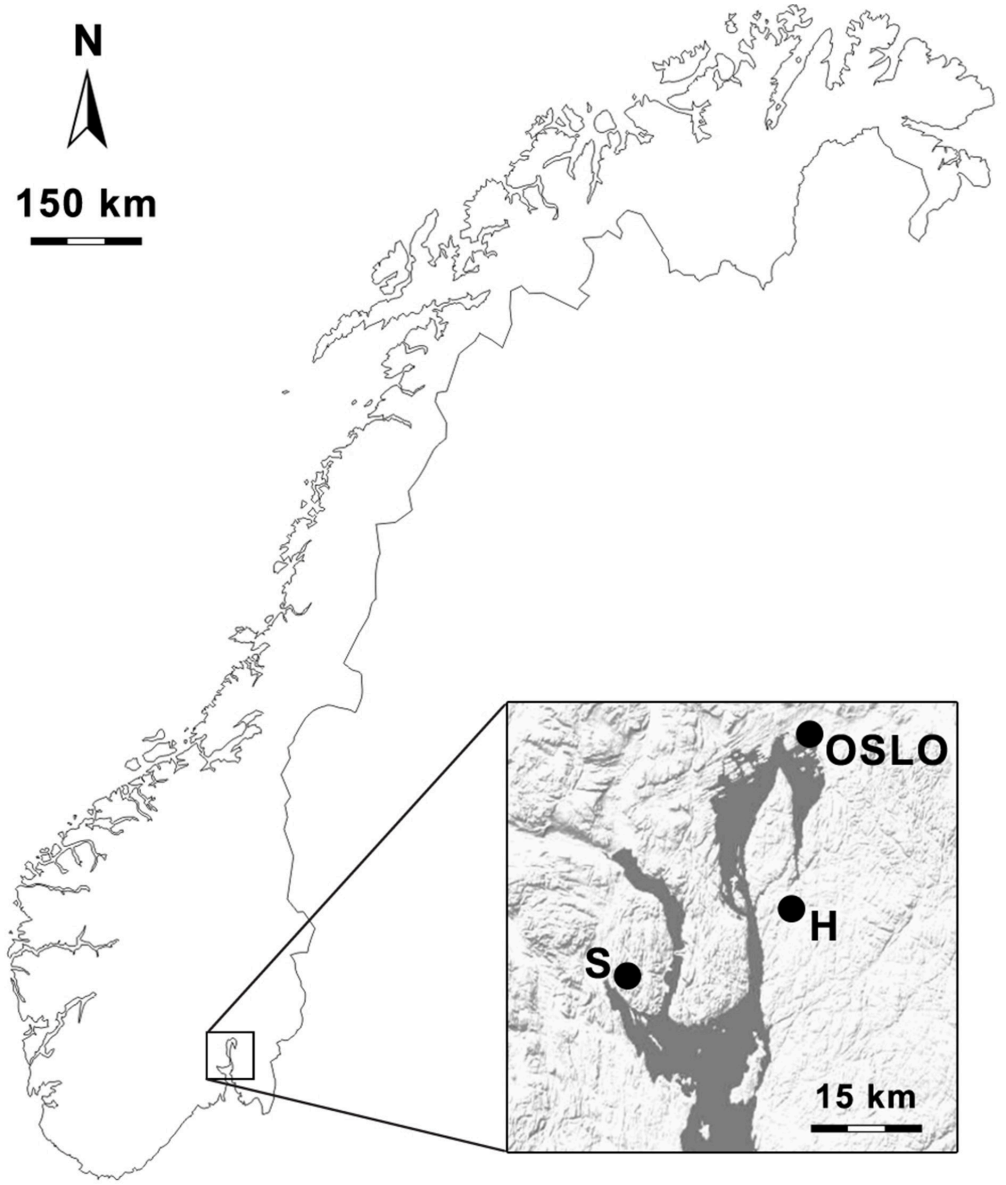
**Map of Norway with the location of both study sites, Sande (S) and Hoxmark (H) in southern Norway**.

We studied Norway spruce [*Picea abies* (L.) Karst.] trees at two forest sites; Sande and Hoxmark (Table 1, Figure 1) in southern Norway where scattered individual trees showed visual signs of top dieback. Both sites were at low altitude (80 m–110 m a.s.l.) and had rather shallow soils (44–52 cm) on bare rock with marine sediment. Both sites had high clay content (21–24%) relative to sites sampled systematically across the forest area in Norway. Trees were planted on former agricultural sites about 50 years ago. Both sites have been subjected to common forest practices in Norway; the spruce is planted, and this has been followed by weed and grass removal around plants, later by pre-commercial thinning and removal of most of the naturally regenerated broadleaves, and finally thinning and occasional removing of dead trees. The sites were well stocked with living trees with 350–400 m^3^/ha, about 900 stems/ha. About 70–80 m^3^/ha dead trees were recorded (300 trees/ha) on each site. Temperature and precipitation records were available from meteorological stations near both study sites. “Potential water deficit” (mm), i.e., a parameter for water availability, was derived from the cumulative precipitation subtracted by the modeled cumulative potential evapotranspiration including parameters of relative humidity, temperature, cloud cover, and wind speed as input variables. More details about the calculation method and about the study sites can be found in Hentschel et al. (2014). Information about the mean monthly precipitation and the daily water deficit is available in Supplement Figure 1.

**Table 1:** **Information on study sites and growth parameters (mean ± SE) of Norway spruce trees harvested in southern Norway**.

**Site**	**Lat. (N)**	**Long. (E)**	**Health state**	**DBH 2011 (cm)**	**Tree height 2001 (cm)**	**Tree height 2011 (cm)**	**N**
Sande	59°35’12”	10°12’30”	Healthy	30.1 ± 1.8	1635.5 ± 47.4	2193.5 ± 56.4	6
			Symptomatic	30.8 ± 2.1	1596.5 ± 51.5	2043.1 ± 43.2	6
Hoxmark	59°40’19”	10°45’11”	Healthy	27.2 ± 1.3	1813.8 ± 43.2	2308.2 ± 46.8	6
			Symptomatic	28.5 ± 1.8	1898.5 ± 32.8	2212.3 ± 57.9	6

*No significant differences in breast height diameter (DBH) or tree heights between healthy looking and trees with top dieback were found*.

**Figure 2:**
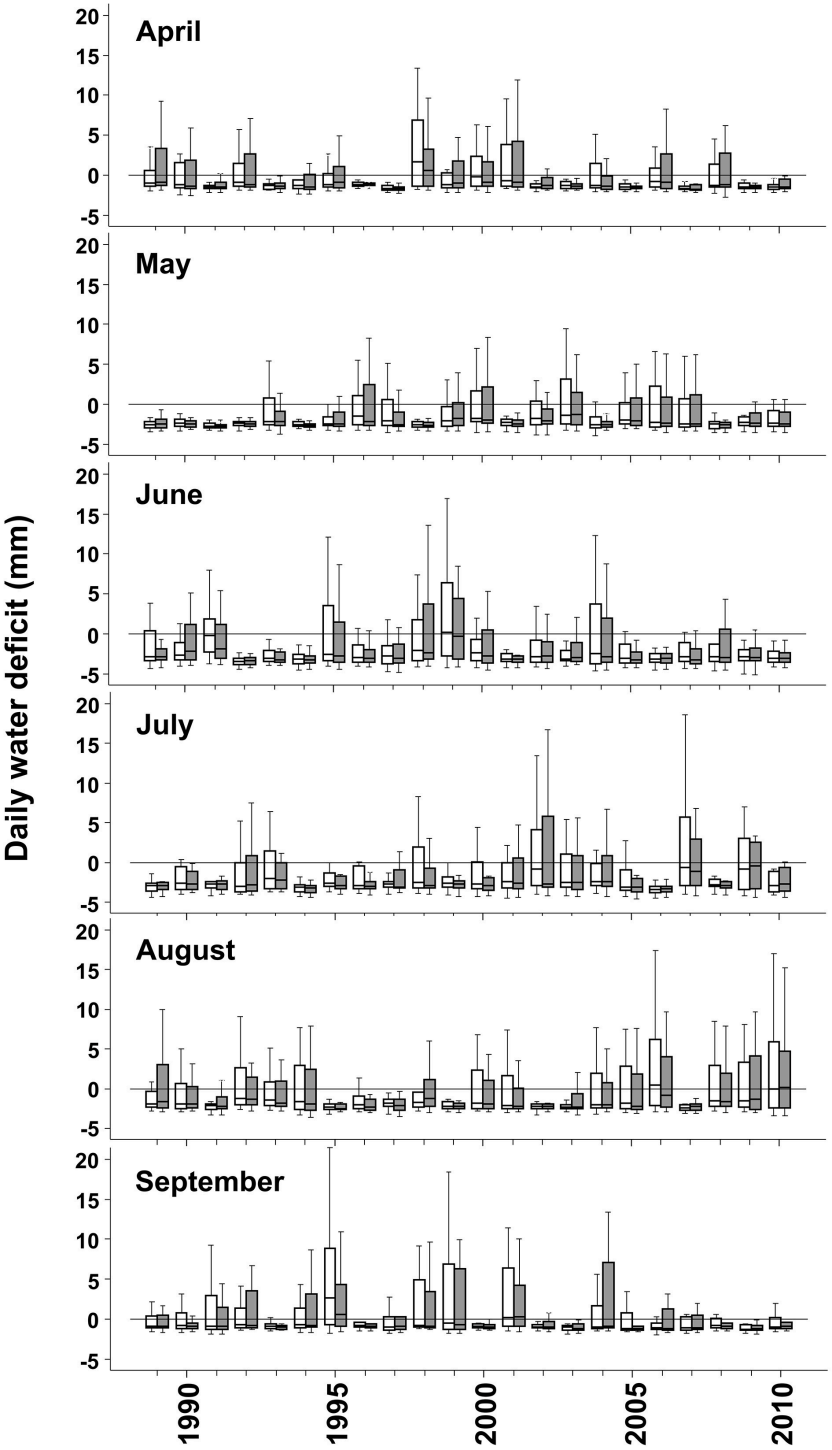
**Daily water deficit (mm) in Sande (empty bars) and Hoxmark (gray filled bars) during the vegetation period (April–September) from years 1989 until 2010**. Box- and whiskers plots give information on the highest and the lowest observation, the upper and the lower quartile and the median.

We selected 10 Norway spruce tree pairs at Sande and Hoxmark, respectively. Each pair consisted of one tree with dieback symptoms, i.e., needle yellowing, needle shortening and decrease in height increment, and the nearest healthy looking neighbor. We use the terminology “healthy looking” because trees with no visual symptoms may have lower sap flux density than expected (Børja et al., 2016). The trees were 40–50 years old, and all sampled trees were still living. Six tree pairs were harvested on each study site in September 2011. Information on growth characteristics of these trees can be found in Table 1. Diameter at breast height showed no significant differences across sites and between healthy looking and declining trees, thus no influence on wood structure due to cambial maturation was projected (Lundgren, 2004). For that reason, and because the two sites showed quite similar soil characteristics as well as daily water deficits (Figure 2), a pooled analysis for annual variability of anatomical traits was carried out. Wood boles (25 cm) were cut of the 10th whorl from the top immediately after harvesting. After de-barking in the field, boles were transported to the lab in plastic bags containing some fresh water and containing 0.01 vol. % Micropur (Katadyn Products Inc.). From all 40 trees, wood cores (13 mm) were taken at breast height in order to perform anatomical analyses with SilviScan technology. Sapwood specimen for determination of *P*_50_, i.e., an outer sapwood zone of 20 mm separated from the wood boles by means of a chisel, as well as wood cores were stored frozen (−18°C) until further preparation steps.

### Vulnerability to cavitation (*P*_50_)

The dataset for calculating *P*_50_ values of selected sapwood specimen was available from a previous study (Rosner et al., 2014). *P*_50_ was defined as the positive pressure inducing 50 % loss of hydraulic conductivity, also termed “air-seeding pressure.” The method to measure *P*_50_ on small trunkwood beams by means of the pressure collar technique is described in Domec and Gartner (2002a) and Rosner et al. (2008). We isolated outer sapwood specimens with a transverse surface area of about 9 × 9 mm^2^ by splitting the wood along the grain with a chisel. Thereafter, small wood beams with tangential and radial dimensions of 6 mm, respectively, were produced on a sliding microtome. Samples were shortened to 130 mm on a band saw and ends were re-cut with a razor blade to a final length of 120 mm. Specimens had to be kept wet during all preparation steps. Wood beams were thereafter soaked in distilled water under partial vacuum for at least 48 h to refill embolized tracheids. Hydraulic conductivity measurements with distilled, filtered (0.22 μm), and degassed water containing 0.005 vol. % Micropur were carried out under a pressure head of 5.4 kPa (54 cm water column). After measurement of the conductivity at full saturation, air overpressure was applied to the specimens by means of a double-ended pressure chamber (PMS Instruments Co., Corvallis, Oregon). Hydraulic conductivity was measured again after a relaxation period in distilled water of 30 min. Initially, the pressure chamber was pressurized to 1.0 MPa, and the pressure was thereafter subsequently increased after each conductivity measurement in steps of 0.5–1.0 MPa until more than 95% loss of conductivity was reached. **P**_50_ values for each sapwood beam were calculated as described in Pammenter and Vander Willigen (1998). In most of the specimen, conductivity measurements were however performed only until 70% loss of hydraulic conductivity, since that proved sufficient to calculate reliable *P*_50_ values for each tree segment (Rosner et al., 2014). In general, the **P**_50_ value for a tree segment is calculated from pooled conductivity and pressure data of more than three single wood beams. It is thus not necessary, that for each wood beam a complete vulnerability curve, i.e., the conductivity loss plotted against the positive pressure, is available. For the present study, selected wood beams were used, where (a) annual rings were perfectly parallel aligned to the tangential longitudinal surfaces, where (b) complete annual rings were present and where (c) a *P*_50_ value could be calculated, thus where data for a complete vulnerability curve were available (*n* = 19). A detailed description of the origin of these specimens can be found in Supplement Table 1.

### Anatomical investigations with SilviScan technology and dataset of potential proxies

From the selected wood beams, small wood cubes with radial, tangential and longitudinal dimensions of 6 mm were sawn. Wood cores were thawed and soaked in 96% ethanol in order to avoid the development of cracks or deformation due to shrinkage processes during the drying process at ambient temperature. From dry wood cores and cubes, strips with longitudinal dimension of 7 mm and tangential dimension of 2 mm were sawn by a twin-blade saw. Wood strips were then analyzed by SilviScan technology at CSIRO Forestry and Forest Products (Australia) (Evans, 1994, 1999). X-ray microdensity, radial and tangential tracheid diameters (lumen diameter plus single cell wall, thus the dimension from one to the next middle lamella) and cell wall thickness were assessed in 25 μm radial measurement steps. After cross-dating the wood cores based on the X-ray microdensity variations, a dataset of ring widths (RW) and potential functional traits for each annual ring from 1990 to 2010 was calculated. The same dataset was created for the wood cubes.

**Figure 3:**
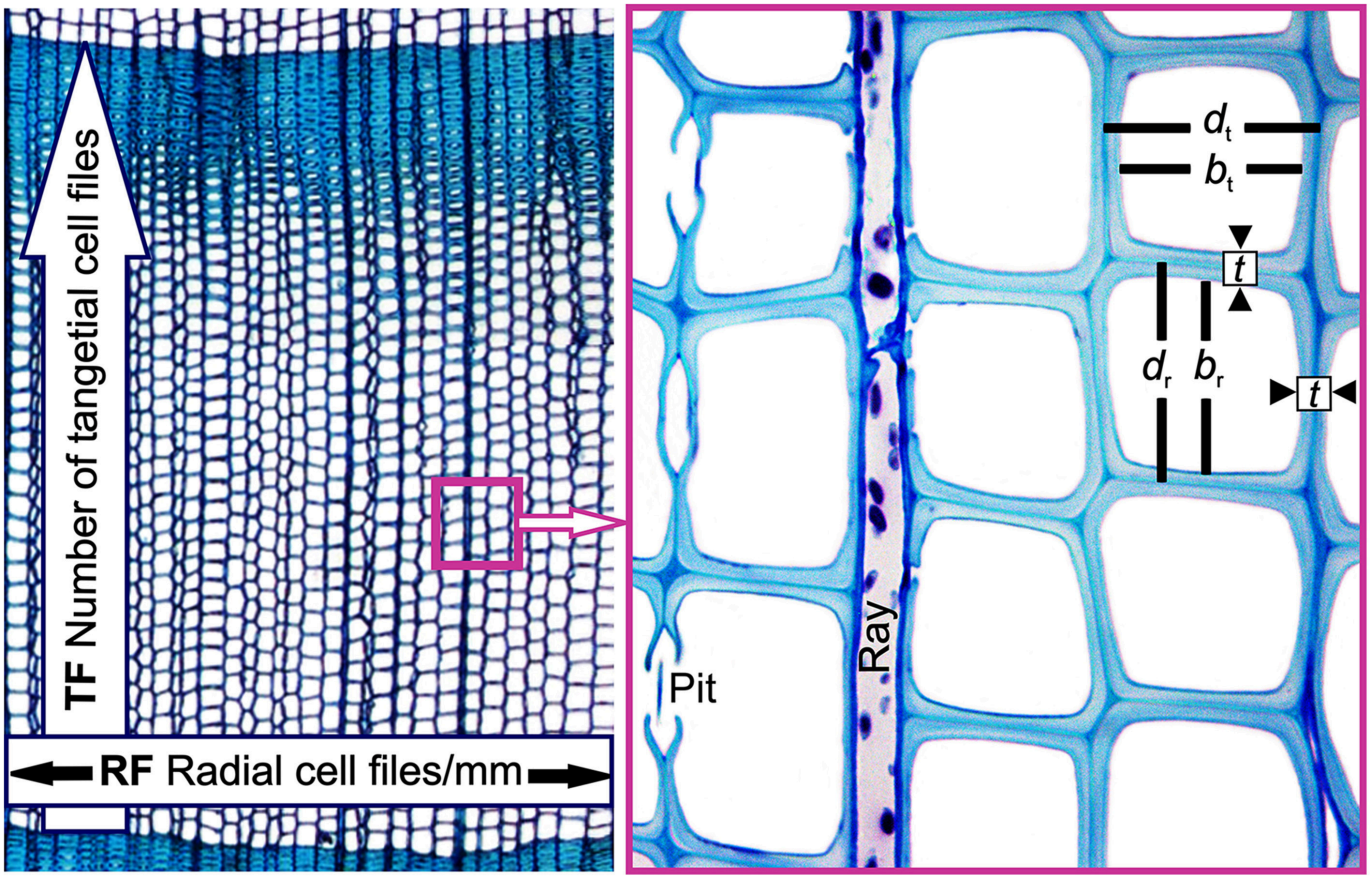
**Visual description of selected anatomical traits: Theoretical number of radial tracheid files/mm circumference (RF) is defined as 1000 μm/mean tangential cell diameter (*d*_*t*_, i.e., the mean tangential distance from one to the next middle lamella in μm)**. The theoretical number of tangential tracheid files/annual ring (TF) is calculated from the mean radial cell diameter (*d*_*r*_, i.e., the mean radial distance between two middle lamellae). Shown are also the radial lumen diameter (*b*_r_), tangential lumen diameter (*b*_*t*_), and the radial and tangential double wall thickness (*t*).

Theoretical number of radial tracheid files of 1 mm circumference was estimated as: RF = (1000 μm/mean tangential cell diameter (*d*_t_, i.e., the mean tangential lumen diameter plus single cell wall thickness in μm, Figure 3). We defined the theoretical number of tangential tracheid files/annual ring as TF = RW (in μm)/mean radial cell diameter (*d*_r_, i.e., radial lumen diameter plus single cell wall thickness in μm, Figure 3).

Hydraulic lumen diameters were calculated as Σ*D*^5^/Σ*D*^4^, where *D* is the individual tracheid lumen diameter. The method is preferable over other artificial methods for hydraulically weighting diameter distributions (Kolb and Sperry, 1999). Tracheids of this diameter should cavitate at *P*_50_ in case that air-seeding progresses from wide to narrow tracheids (Hacke et al., 2001). Hydraulic lumen diameters were derived for mean lumen dimensions, i.e., the mean value of radial and tangential lumen diameters, (*b*_h_), for radial lumen dimensions (*b*_hr_) and for tangential lumen dimensions (*b*_ht_).

The conduit wall reinforcement is estimated as (*t*/*b*)^2^, where *t* is the cell double wall thickness and *b* is the lumen diameter (Hacke et al., 2001). (*t*/*b*)^2^ was calculated for tracheids with diameters ±10% of *b*_h_ (Pitterman et al., 2006; Domec et al., 2009). Using a percent value is applicable across different species, since ± μm suggestions are restricted not only to a given species but also to a specific organ (needles, branch, trunk, roots) within a tree, because lumen diameters are highly variable within a trunk (Anfodillo et al., 2006, 2013; Domec et al., 2009; Carrer et al., 2015). According to different hydraulic lumen diameters presented in this study, (*t*/*b*)^2^ was estimated for ±10% of *b*_h_ ((*t*/*b*_h_)^2^), for ± 10% of *b*_hr_ ((*t*/*b*_hr_)^2^), and for ± 10% of *b*_ht_ ((*t*/*b*_ht_)^2^).

### Statistical analyses and numbers of samples

Data are given as mean ± standard error (SE) or as box and whiskers plots. The Pearson's correlation coefficient was used to test the associations between traits. Mean values were examined for significant differences by the Student's *t*-test. Data were tested for normal distribution with the Kolmogorov-Smirnov test. Relationships between traits and differences in mean values were accepted as significant if P was <0.05. Analysis was carried out with SPSS® 21.0.

The relationship between *P*_50_, ring width, cell dimensions and potential hydraulic proxies was tested on 19 sapwood specimens originating from 12 trees (Supplement Table 1). Chronologies of ring width and selected anatomical traits from 1990 until 2010 were analyzed in 40 trees. For comparisons between healthy looking and trees with top dieback symptoms, mean tree data of the two sites were pooled. Thus, mean values of 20 healthy looking trees and 20 trees with top dieback could be statistically compared (Student's *t*-test). For anatomy-climate relations of years 1990–2010 (21 years), mean monthly temperature and precipitation were related to annual means of ring widths and selected anatomical parameters at Sande and Hoxmark sites, respectively. This means that each correlation analysis for healthy looking trees and trees with top dieback symptoms was performed with a set of 42 data pairs (21 years of two sites). Correlations for mean daily water deficit resembled those for precipitation, and are thus not shown.

## Results

### Relationship between *P*_50_, growth and anatomical traits

**Figure 4:**
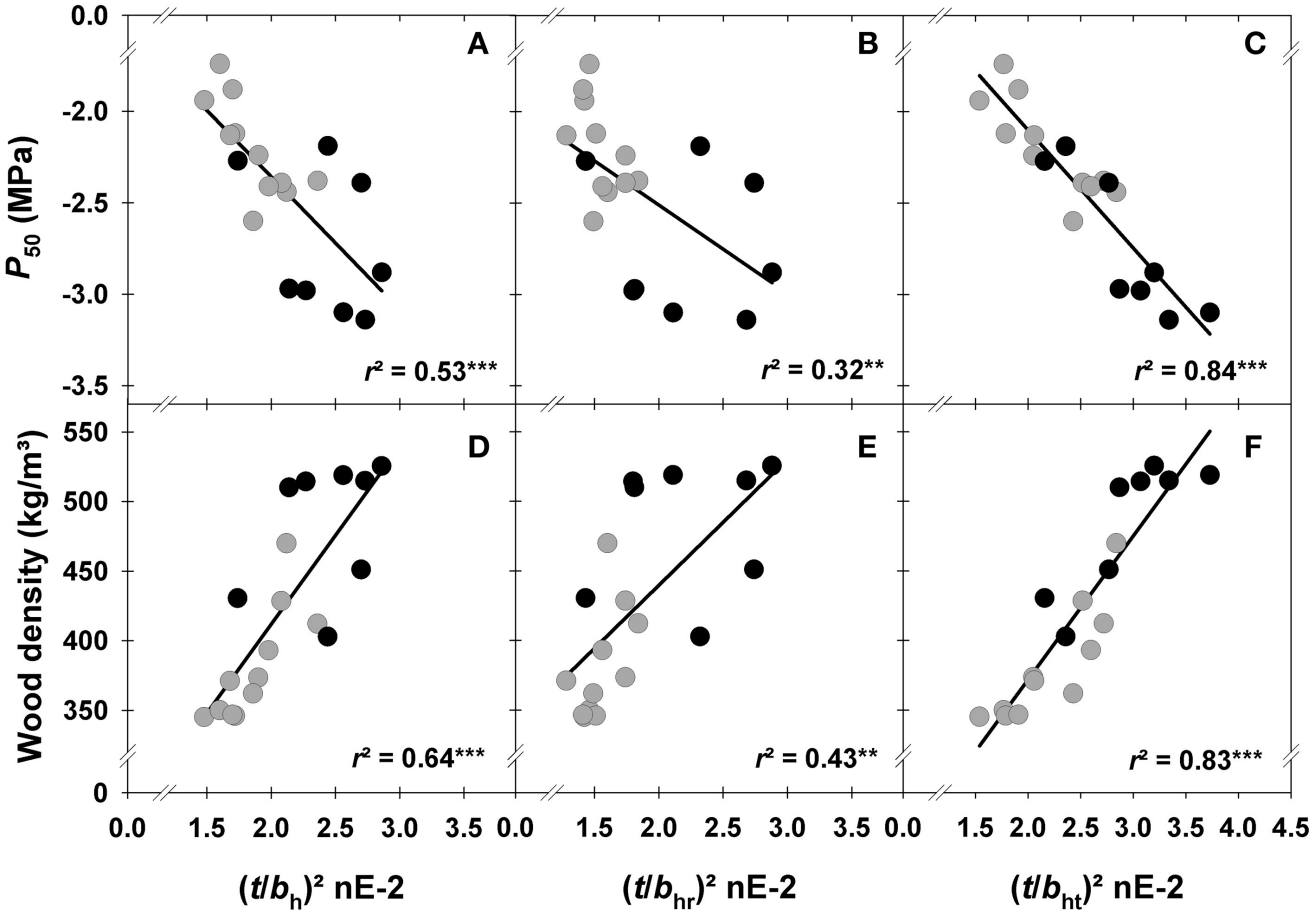
**Vulnerability to cavitation (*P*_50_) and wood density plotted against conduit wall reinforcement parameters of tracheids with ±10% of the hydraulic lumen diameter ((*t*/*b*_*h*_)^2^; A,D), with ±10% of the radial hydraulic lumen diameter ((*t*/*b*_*hr*_)^2^; B,E), and with ±10% of the tangential hydraulic lumen diameter ((*t*/*b*_*ht*_)^2^; C,F)**. Gray dots indicate samples from healthy looking trees, black dots from trees with top dieback. Significant relationships at the 1% level are indicated with ** and at the 0.1% level indicated with ***.

The outermost still functional sapwood of trees with top dieback symptoms was characterized by a higher hydraulic safety than that of healthy looking trees (Figures 4A–C). At the time of harvesting, vulnerability to cavitation (*P*_50_) was thus significantly higher in specimen of trees with wider annual rings (Table 2). Surprisingly, tangential lumen diameters (*b*_t_) were a better proxy for *P*_50_ than radial lumen diameters (*b*_r_). The theoretical number of radial tracheid files (RF), which depends on tangential lumen diameters, was thus tighter correlated with *P*_50_ than the number of tangential files (TF). The hydraulic lumen diameter (*b*_h_) was a good predictive trait for *P*_50_, however, the hydraulic lumen diameter calculated from radial cell dimensions (*b*_*hr*_) was much weaker related to *P*_50_ than the tangential hydraulic lumen diameter (*b*_ht_). Double cell wall thickness was tightly correlated with *P*_50_ (Table 2). The conduit wall reinforcement of tracheids with ±10% of the hydraulic diameter ((*t*/*b*_h_)^2^) was therefore strongly related to *P*_50_ as well (Figure 4A). The best proxy for *P*_50_ was the conduit wall reinforcement derived from the tangential hydraulic lumen diameter ((*t*/*b*_ht_)^2^), whereas the correlation between *P*_50_ and (*t*/*b*_hr_)^2^, was much weaker (Table 2, Figures 4B,C). (*t*/*b*_ht_)^2^ was stronger related to mean ring wood density (Figure 4F) than (*t*/*b*_h_)^2^ (Figure 4D) or (*t*/*b*_hr_)^2^ (Figure 4E). To sum up, conduit wall reinforcement estimated from the mean hydraulic lumen diameter ((*t*/*b*_h_)^2^ was a quite suitable trait for predicting *P*_50_ but (*t*/*b*_ht_)^2^ was even better (Figure 4C).

**Table 2:** **Relationships between *P*_50_, growth and anatomical traits on selected wood beams (*n* = 19, description in Supplement Table 1)**.

	**RW**	**RF**	**TF**	***b_*r*_***	***b_*t*_***	***t***	***b_*h*_***	***b_*hr*_***	***b_*ht*_***	**(*t*/*b_*h*_*)^2^**	**(*t*/*b_*hr*_*)^2^**	**(*t*/*b_*ht*_*)^2^**
*P*_50_	0.474^*^	−0.655^**^	0.440	0.629^**^	0.788^***^	−0.886^***^	0.668^**^	0.453	0.639^**^	−0.730^***^	−0.567^**^	−0.917^***^
RW		−0.396	0.984^***^	0.753^***^	0.474^*^	−0.539^*^	0.643^**^	0.624^**^	0.364	−0.366	−0.277	−0.484^*^
RF			–0.326	−0.607^**^	−0.970^***^	0.517^*^	−0.793^***^	−0.428	−0.955^***^	0.387	0.183	0.659^**^
TF				0.635^**^	0.404	−0.496^*^	0.522^*^	0.488^*^	0.325	−0.303	−0.193	−0.449
*b_*r*_*					0.694^***^	−0.697^***^	0.946^***^	0.945^***^	0.513^*^	−0.633^**^	−0.609^**^	−0.654^**^
*b_*t*_*						−0.707^***^	0.844^***^	0.499^*^	0.943^***^	−0.559^*^	−0.351	−0.797^***^
*t*							−0.681^***^	−0.527^*^	−0.552^*^	0.857^***^	0.717^***^	0.917^***^
*b_*h*_*								0.873^***^	0.721^***^	−0.663^**^	−0.583^**^	−0.715^***^
*b_*hr*_*									0.294	−0.595^**^	−0.666^**^	−0.485^*^
*b_*ht*_*										−0.415	−0.156	−0.689^***^
(*t*/*b_*h*_*)^2^											0.931^***^	0.859^***^
(*t*/*b_*hr*_*)^2^												0.661^**^

The significance of the Pearson's correlation coefficients are indexed with

* if P < 0.05, with

** if P < 0.01, and with

*** *if P < 0.001*.

*Full names of the traits can be found in the list of Abbreviations. Statistically significant correlations are also indicated by bold fonts*.

### Water deficit in years studied (1990–2010)

The most extreme year during the study period was 1992, where mean daily water deficit and temperature was very high in June (Figure 2, Supplement Figures 1, 2A). Mean September precipitation gradually decreased from 1991 until 1993 (Supplement Figure 2B). In 1994, during the whole early summer, daily water deficit was relatively high; August was however quite wet (Supplement Figure 2C). Until 2004, no climate extremes could be found; except in 2001, where June was relatively dry. 2005 and 2006 were characterized by dry June and July months but also by drier September months compared to the previous years. 2007 was a year with very high water supply, and can be considered as an extreme year (Supplement Figure 1).

### Climatic sensitivity of hydraulic traits in healthy looking trees and trees with top dieback symptoms

**Figure 5:**
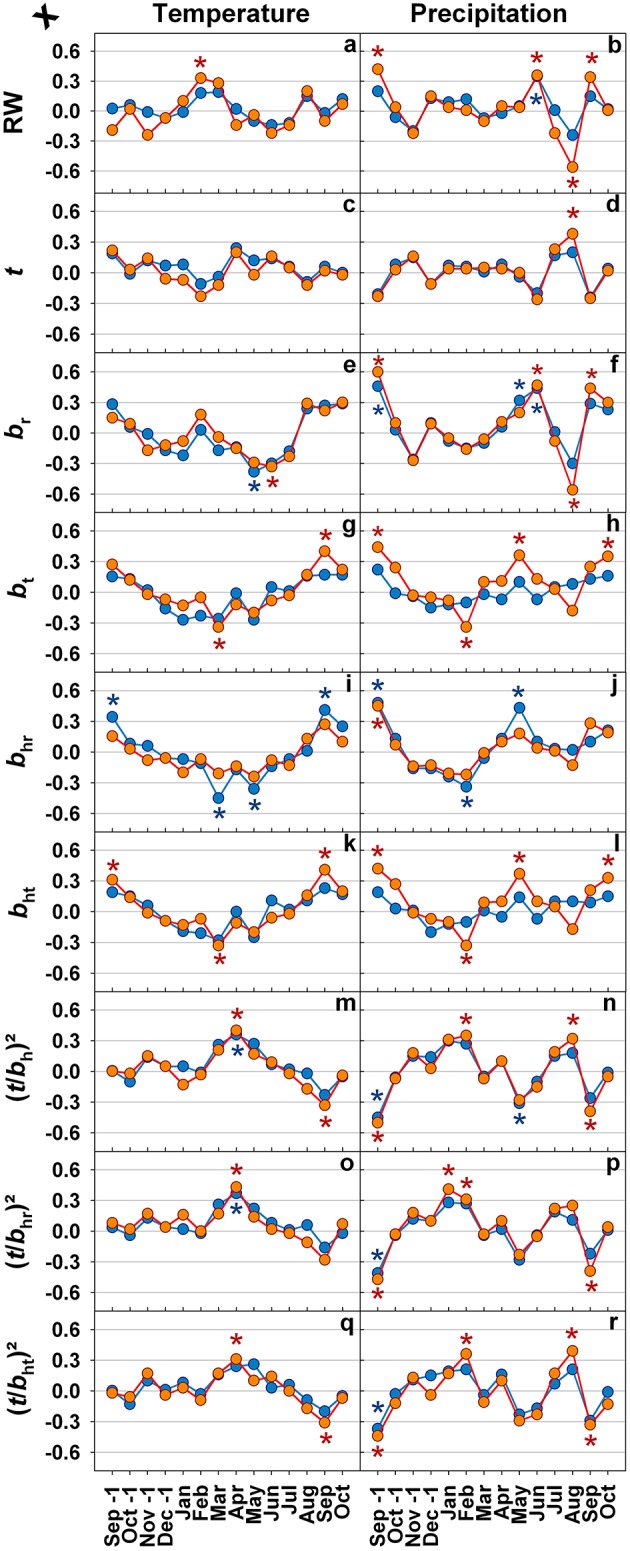
Pearson's correlation coefficients for monthly mean temperature as well as precipitation from September of the previous growing season until current years October and ring width (RW; A,B), double cell wall thickness (*t*; C,D), mean radial lumen diameter (*b*_*r*_; E,F), mean tangential lumen diameter (*b*_*t*_; G,H), mean radial hydraulic lumen diameter (*b*_*hr*_; I,J), mean tangential hydraulic lumen diameter (*b*_*ht*_; K,L), conduit wall reinforcement based on the hydraulic lumen diameter ((*t*/*b*_*h*_)^2^; M,N), conduit wall reinforcement based on the radial hydraulic lumen diameter ((*t*/*b*_*hr*_)^2^; O,P), and conduit wall reinforcement based on the tangential hydraulic lumen diameter ((*t*/*b*_*ht*_)^2^; Q,R) for the period between 1990 and 2010. Blue lines and symbols represent healthy looking trees, red lines and symbols trees with symptoms of top dieback. Significant correlations at (least at) the 5% level are indicated with * in blue for healthy looking trees and * in red for trees with symptoms of top dieback.

In Figure 5 correlations for mean monthly temperature and precipitation, annual radial growth and selected anatomical traits are given. Climatic sensitivity of ring width and anatomical traits differed marginally between healthy looking trees and trees with top dieback. Temperature had almost no effect on ring width (Figure 5A), whereas high precipitation in June had a positive impact on annual increment (Figure 5B). Cell wall thickness was not related to temperature (Figure 5C) and only slightly positively to precipitation in August (Figure 5D). Radial lumen diameters were positively influenced by high precipitation in September of the previous growing season and in June (Figure 5F) rather than by temperature (Figure 5E). Only for trees with top dieback symptoms significant influence of temperature (Figures 5G,K) and precipitation (Figures 5H,L) on tangential (hydraulic) lumen diameters were found. In both tree groups, radial hydraulic lumen diameters responded significantly positively to high precipitation in September of the previous growing season (Figure 5J) but weakly to temperature (Figure 5I). Conduit wall reinforcement traits had rather weak relationships with temperature; higher temperature in April resulted however in a slight increase in (*t*/*b*)^2^ (Figures 5M,O,Q). In both tree groups, (*t*/*b*)^2^ traits responded similarly to changes in temperature and precipitation, where September precipitation had the most significant impact on conduit wall reinforcement (Figures 5N,P,R).

### Chronologies of ring width and anatomical traits in healthy looking trees and trees with top dieback symptoms

**Figure 6:**
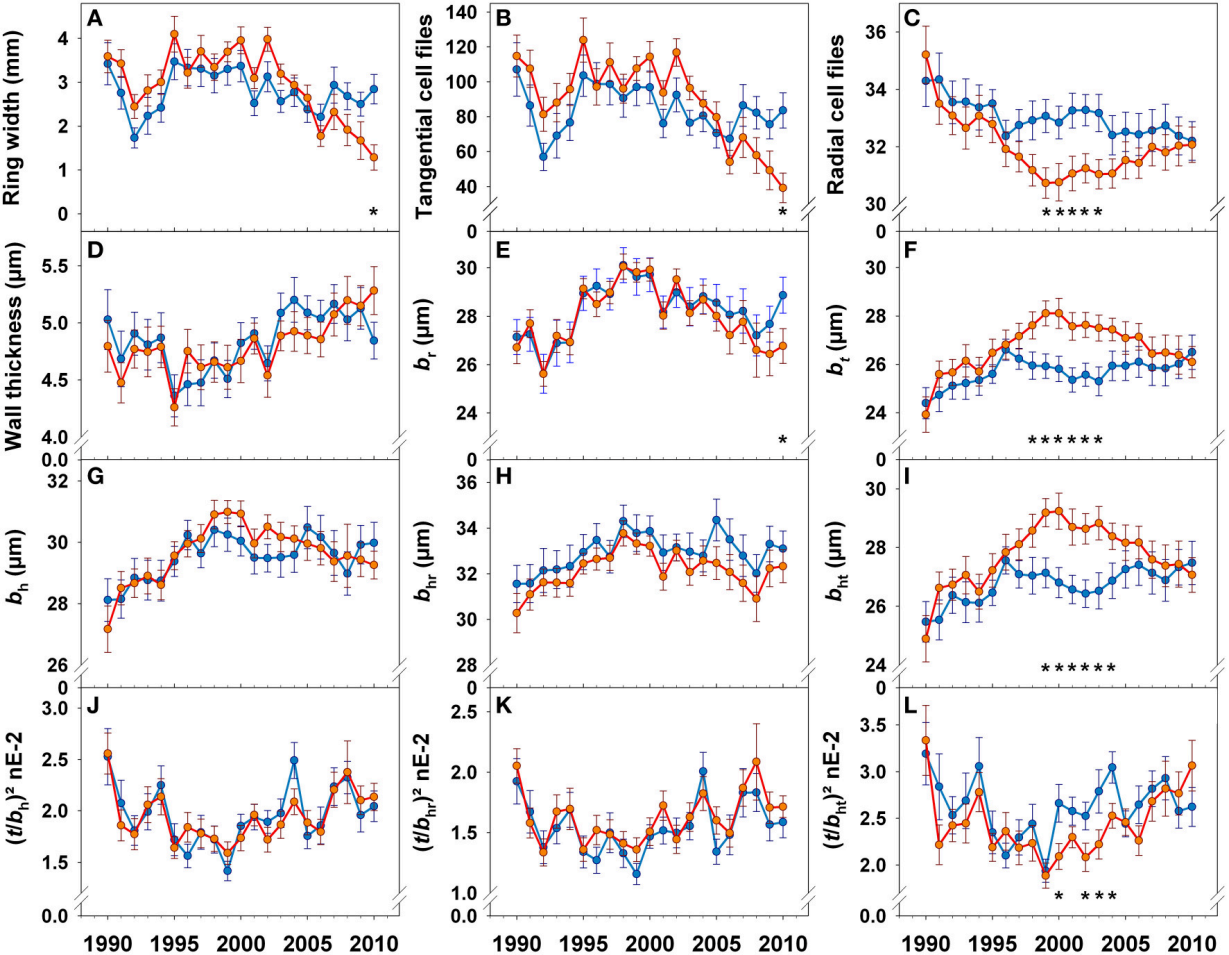
**Chronologies of ring width (A), tangential cell files (B), radial cell files/mm (C), double cell wall thickness (D), mean radial lumen diameter (*b*_*r*_, E), mean tangential lumen diameter (*b*_*t*_, F), mean hydraulic lumen diameter (*b*_*h*_, G), mean radial hydraulic lumen diameter (*b*_*hr*_, H), mean tangential hydraulic lumen diameter (*b*_*ht*_, I), conduit wall reinforcement based on the hydraulic lumen diameter ((*t*/*b*_*h*_)^2^, J), conduit wall reinforcement based on the radial hydraulic lumen diameter ((*t*/*b*_*hr*_)^2^, K), and conduit wall reinforcement based on the tangential hydraulic lumen diameter ((*t*/*b*_*ht*_)^2^, L) for the period between 1990 and 2010**. Blue lines and symbols represent healthy looking trees, red lines and symbols trees with symptoms of top dieback. Significant differences between tree groups (healthy looking and trees with dieback symptoms) at (least at) the 5% level are indicated with *.

The year 1992, where early summer was extremely dry (Supplement Figure 1), was characterized by a sudden decrease in ring width (Figure 6A) due to a lower number of tangential tracheid files (Figure 6B) and mean radial lumen diameters had minimum peak (Figure 6E). Although, 1994 was a year with high summer water deficit, ring width increased gradually until 1995. A slight decrease in ring width as well as in radial lumen diameter (Figure 6E) was present in 2001. Ring width in trees with top dieback was slightly, but not significantly, higher than in healthy looking trees in almost all annual rings formed before 2004 (Figure 6A). After 2005, ring width showed a different trend in trees with top dieback: annual increment decreased gradually and was significantly lower in 2010 than in healthy looking trees. In 2010, mean radial lumen diameter was therefore significantly smaller in trees with top dieback (Figure 6E). However, 2007, the year with the highest water supply of the investigation period (Supplement Figure 1, Figure 2), was characterized by a slight increase in ring width in both tree groups.

Whereas annual fluctuations and mean values of radial (Figures 6E), radial hydraulic (Figure 6H) and mean hydraulic lumen diameters (Figure 6G) were quite similar until 2009 in both tree groups, chronologies of tangential lumen diameters (Figure 6F) and the number of radial cell files (Figure 6C) exhibited totally different trends after 1996. Between 1998 and 2002, trees with top dieback produced tracheids with significantly wider tangential lumen diameters than healthy looking trees (Figure 6F). Tangential hydraulic lumen diameters were significantly larger (Figure 6I) and the number of radial cell files significantly lower (Figure 6C) in trees with top dieback in the period from 1999 until 2003. Wall thickness showed no significant differences between both tree groups (Figure 6D). Therefore, the (*t*/*b*)^2^ based on the mean hydraulic lumen diameter and on the radial lumen diameter showed similar trends in both tree groups (Figures 6J,K), whereas (*t*/*b*)^2^ derived from tangential lumen diameters was significantly lower in trees with top dieback between 2000 and 2004 (Figure 6L) than in healthy looking trees.

## Discussion

### Conduit wall reinforcement: focus on tangential tracheid diameter

The best proxy for *P*_50_ was (*t*/*b*_ht_)^2^, the conduit wall reinforcement based on tangential hydraulic lumen diameters (Figure 4C). The parameter (*t*/*b*_h_)^2^, i.e., the second power of the wall (*t*) to span (*b*) ratio of tracheids which show little deviation from a calculated hydraulic lumen diameter, was introduced by Hacke et al. (2001) as an estimate for *P*_50_. Lower vulnerability to cavitation implies the need for a safer cell design with either smaller lumen or thicker walls to resist implosion, because the cell walls have to withstand higher negative xylem pressures before cavitation occurs. Several approaches to calculate this parameter and hydraulic lumen diameters have been presented in the literature so far. Radial dimensions (e.g., Domec et al., 2009; Hereş et al., 2014; Wilkinson et al., 2015), both radial and tangential dimensions (e.g., Mencuccini et al., 1997; Jyske and Hölttä, 2015) or tracheid diameter modeled from lumen area measurements, considering the lumen either to be rectangular or circular (Mayr and Cochard, 2003; Anfodillo et al., 2006) have been used for calculating hydraulic diameters. Tracheid lumen and wall thickness measurements for *t*/*b* or (*t*/*b*)^2^ are either performed in the first tangential files of earlywood (Rosner et al., 2009), in whole earlywood, since it accounts for most of the hydraulic conductance (Bouche et al., 2014; Rosner et al., 2016), or in several radial files across the whole annual ring (Hacke et al., 2001; Mayr and Cochard, 2003; Domec et al., 2009; Hacke and Jansen, 2009; Hereş et al., 2014). In the latter case, first a hydraulic diameter is calculated based on the assumption that tracheids with this given diameter cavitate right at *P*_50_. Thereafter, (*t*/*b*_h_)^2^ is assessed for all tracheids with e.g., ±10% (Domec et al., 2009) of the hydraulic diameter.

In Norway spruce, *P*_50_ is strongly related to wood density across cambial age (Rosner et al., 2014). In the present study, we found that (*t*/*b*_ht_)^2^ was much stronger related to wood density than (*t*/*b*_hr_)^2^ (Figures 4E,F); (*t*/*b*_h_)^2^ is calculated as a mean of radial and tangential tracheid lumen dimensions and holds thus an intermediate position (Figure 4D). We do not have a sound explanation for this result yet, since studies on tangential lumen diameter changes within an annual ring (Vysotskaya and Vaganov, 1989) or with cambial age (Lundgren, 2004; Keunecke et al., 2009) are rather scarce and, moreover, no studies on the predictive quality for *P*_50_ exist. There is however no doubt, that radial tracheid diameters are highly variable within an annual ring (earlywood and latewood) and they are more influenced by climate and cambial age than tangential lumen diameters, because the number of periclinal (radial: inside to outside) division is much higher than that of anticlinal (circumference: side) division (Vysotskaya and Vaganov, 1989; Larson, 1994; Keunecke et al., 2009). The stability of this trait indicated by its normal distribution within a conifer annual ring (Vysotskaya and Vaganov, 1989) might be an advantage regarding the predictive quality for *P*_50_. Since (*t*/*b*_*hr*_)^2^ traits were calculated from tracheids with a mean hydraulic diameter, a masking effect of (hydraulically more sensitive but extremely dense) latewood (Domec and Gartner, 2002b; Rosner, 2013; Dalla-Salda et al., 2014) can be excluded. In the next sections, the climate dependence of the tangential (hydraulic) lumen diameter and (*t*/*b*_ht_)^2^ as well as the variability of these traits in healthy looking trees and in trees with top dieback is discussed.

### Annual variation of hydraulic vulnerability is triggered by climate

Ring width was slightly and positively related to spring temperatures (Churakova et al., 2014), but also to precipitation in September of the previous growing season and in current June (Figures 5A,B). In accordance, Mäkinen et al. (2002) found that precipitation during the growing season at low altitude sites (130 m a.s.l.) in southern Norway is positively correlated to growth. At lower latitude sites, in southern Germany, high amounts of precipitation in July were found to foster growth of Norway spruce (Zang et al., 2012). The limiting effect of low precipitation on Norway spruce growth decreases, however the effect of temperature increases with increasing latitude or altitude (Jyske et al., 2014). At low and intermediate elevation sites in the Italian Alps, median cell lumen area of Norway spruce annual rings benefited from August, September and October precipitation of the previous growing season and from current year June precipitation (Castagneri et al., 2015). Radial lumen diameters were positively related to high precipitation (thus low temperature) in May and June (Figures 5E,F). Similar results are reported by Gričar et al. (2015) for Norway spruce growing in Slovenia and Czech Republic: lumen dimension of earlywood tracheids are positively affected by precipitation in the previous autumn and early summer of the current growing season. The significant negative correlation we found between precipitation in August and ring width as well as radial lumen dimensions is difficult to explain logically, since at this time of the year cell division has ceased already (Mäkinen et al., 2003; Rossi et al., 2008, 2013; Henttonen et al., 2009; Jyske et al., 2014). An explanation may be that dry early summer periods were often followed by quite wet late summers in the period investigated (Figure 2, Supplement Figure 2). In trees with top dieback symptoms, higher precipitation in September of the previous growing season and in May of the current season resulted in larger tangential (hydraulic) tracheid diameters (Figure 5L). Surprisingly, similar relationships were found in healthy looking trees only for the radial hydraulic diameter (Figure 5J).

The most reliable proxy for *P*_50_, (*t*/*b*_ht_)^2^ was negatively correlated to precipitation in September of the previous growing season and current year's May (Figure 5R), this implies, that high water availability in late summer of the previous growing season and in spring triggered the production of annual rings with higher hydraulic vulnerability. For instance, high water deficits in September 1993 and 2003 in combination with May water deficits in 1994 and 2004 (Figure 2) triggered a rapid increase in (*t*/*b*_ht_)^2^ in annual rings 1994 and 2004 (Figure 5I). 1994 was a year with an extremely wet late summer (Figure 1), the outcome was wider annual rings (Figure 6A; Castagneri et al., 2015), tracheids with larger diameters (Figures 6E,F; Gričar et al., 2015) and thinner call walls (Figure 6D) and thus a sudden drop in *(t*/*b*)^2^ traits in 1995 (Figures 6J–L). Stinziano et al. (2015) suggest for boreal forests dominated by Norway spruce, that as the climate warms, the period of net carbon uptake in needles could extend in the autumn, which could increase total carbon uptake in these forests. Warm but not too wet Septembers could result in higher carbon fixation with little impact on the hydraulic vulnerability of the annual ring formed in next growth period.

High temperatures in April had a positive impact on all (*t*/*b*)^2^ parameters. Norway spruce might be thus able to adapt to some extent to warmer spring temperatures that have been observed during the past decades in this region (Mikkonen et al., 2015). Warmer spring temperatures bear however an increased risk of early spring frosts damage due to earlier de-hardening (Schlyter et al., 2006). In trees with top dieback symptoms, *(t*/*b*_ht_)^2^ and cell wall thickness benefited as well from high precipitation in August, however also from low precipitation and temperature in September. Wall thickening in cold climates (e.g., at the treeline) can last until end of August to mid of September (Gindl et al., 2000; Treml et al., 2015), in mild continental temperate climate up to October (Cuny et al., 2012). Wimmer and Grabner (2000) found a positive relationship between mean ring wood density, that was tightly correlated with *(t*/*b*_ht_)^2^ in our study (Figure 4F), and precipitation in August for Norway spruce grown in Germany at low altitudes. Due to the negative relationship between wall thickness and ring width (Table 2) it is however questionable to conclude logically that wet Augusts favor cell wall thickening. Note that dry early summer periods were often followed by wet late summer periods (Supplement Figure 2).

To sum up, climate had a poor effect on cell wall thickness, but quite a strong effect on (hydraulic) tracheid diameters. In both tree groups, high September precipitation in the previous growing season resulted in significantly hydraulically less safe annual rings. Climate-hydraulic proxy relationships shall be tested on other conifer species, since the dynamics of xylogenesis are surprisingly homogeneous among conifer species of the northern hemisphere, although dispersions from the average are observed (Rossi et al., 2013).

### Are trees prone to top dieback opportunists?

We found striking differences in the chronologies of hydraulic proxies between healthy looking and declining tress. However wall thickness and radial (hydraulic) lumen diameters and thus (*t*/*b*_hr_)^2^ did not differ significantly between the two tree groups. On contrary, mean tangential (hydraulic) lumen diameters and (*t*/*b*_ht_)^2^ differed extremely between 2000 and 2004 (Figure 6). During this period, trees with top dieback symptoms invested fewer carbohydrates in hydraulic safety, as indicated by the high predictive quality of *b*_*t*_ and (*t*/*b*_ht_)^2^ for *P*_50_ (Table 2, Figure 4C). Quite unexpected was the strategy of trees with top dieback for producing wider hydraulic lumen diameters; our results suggest that Norway spruce has two opportunities to maintain hydraulic efficiency: either fewer anticlinal cell divisions or enlargement in the radial direction of tracheids produced by periclinal divisions. Fewer anticlinal cell divisions are probably associated with a decreasing trend of annual growth with age (Keunecke et al., 2009) but might be also interpreted as “opportunistic strategy” as it is obviously less costly since trees with top dieback produced wood with lower (*t*/*b*_ht_)^2^ (6L) and thus wood density (Hentschel et al., 2014; Rosner et al., 2014). Climate correlations were found for tangential (hydraulic) lumen in trees with top dieback but not in healthy looking trees. Wall thickness was only marginally affected by climate, therefore, trees with top dieback consequently produced wood with lower (*t*/*b*_ht_)^2^ when e.g., water supply in previous year's September and current May was sufficient. This “opportunistic strategy” can be a risky investment regarding hydraulic safety since it can lead to lower survival prospects under the impact of an extreme sudden drought. Mature Norway spruce needs at least 10 annual rings for axial water transport (Bertaud and Holmbom, 2004). Due to irreversible embolism (Choat et al., 2015) or quite slow recovery from drought induced embolism (reviewed in Zwieniecki and Secchi, 2015), hydraulic conductivity might get lost forever in more vulnerable annual rings or in the most conductive parts of a given annual ring. This can result in an impairment of the water supply of the crown and finally to reduced growth. Healthy looking trees might have been better prepared for the dry July months in 2005/6 (Figure 2), since they produced hydraulically safer wood in the period 2000–2004 as indicated by significantly higher (*t*/*b*_ht_)^2^ (Figure 6L). In accordance, from 2005, annual increment took a different course in trees with top dieback (Figure 6A).

The concept of “opportunistic strategy” is not in line with the more anisohydric behavior (Tardieu and Simonneau, 1998) of trees with top dieback symptoms since they tended to spend water; they had a predisposition to less strict stomatal control (Hentschel et al., 2014). Aguadé et al. (2015) underline the intertwining of physiological mechanisms leading to drought-induced conifer mortality and the difficulty of isolating their contribution under field conditions. According to McDowell et al. (2008), mechanisms that cause mortality in trees are carbon starvation, i.e., failure to maintain metabolism or defense reaction due to prolonged negative carbohydrate balance, and hydraulic failure. Both processes are likely coupled when conifers become vulnerable to pests and extreme climate (McDowell et al., 2016). Anisohydric strategy should be coupled with a more hydraulically safe design in order to avoid conductivity loss during severe droughts when more negative water potentials develop in the tracheids, whereas isohydric behavior, i.e., a more strict control of water loss through stomatal closure, should demand less safe wood (Tardieu and Simonneau, 1998). However, recent studies show that shifts rather than a clear separation between isohydry and anisohydry exist (McDowell, 2011; Klein, 2014; Martínez-Vilalta et al., 2014; Sevanto et al., 2014). Carbon starvation is closely linked to water supply in the plant, since water is necessary for transport of non-structural carbohydrates (carbon reserves) in the secondary phloem (Hartmann et al., 2013a; Sevanto et al., 2014). Whereas catastrophic hydraulic failure in Norway spruce can occur in above-ground tissues, belowground tissues (roots) can die from carbon starvation, indicating that mortality mechanisms are not defined at the organism level but rather within tree compartments (Hartmann et al., 2013b). In accordance, in trees with top dieback, higher fine-root mortality was found (Godbold et al., 2014). The abrupt change from a rather dry period (2005/6) to an extremely wet year in 2007 could have influenced root vitality negatively and could have made trees more vulnerable to biotic agents. Whatever finally caused top dieback, the process was favored by the risky combination of less strict stomatal control (Hentschel et al., 2014) and by the “opportunistic strategy” of producing more vulnerable wood when e.g., precipitation in the previous year's September and in early summer was high.

### Is breeding for higher hydraulic safety possible?

Concerning different, probably genetically determined, wood formation characteristics of healthy looking and declining trees prior to drought stress (under sufficient water supply), the question arises if it was possible to breed Norway spruce or other conifer species for higher hydraulic safety (Dalla-Salda et al., 2011; Montwé et al., 2016). Wood density (Rosner et al., 2014) or (*t*/*b*_ht_)^2^ could be easily applicable screening tools and early selection for wood density is highly effective from rings 6–7 in Norway spruce (Chen et al., 2014). Starting at that age, selecting individuals for higher hydraulic safety in trunkwood could be thus possible.

Trunkwood safety covers however only one aspect of drought sensitivity related to hydraulic architecture (Hacke et al., 2015), and selected trees shall be tested for their hydraulic performance in field experiments. In a recent study on potted 3-year-old seedlings, limited variation at the family level indicates that the response to drought is quite conservative within Norway spruce, which may limit breeding opportunities for increased drought resistance (Chmura et al., 2016). At that young age, hydraulic safety is however anyway very high and hydraulic structure-function relationships (e.g., with density) are masked by mechanical demands of the young trunk (Rosner, 2013). It must be also considered, that in mature Norway spruce wood, density (and thus (*t*/*b*)^2^) is genetically negatively correlated with growth (Hannrup et al., 2004; Rosner et al., 2014). When selecting such conifer species for higher hydraulic safety, reduction in growth might have to be taken into account (Montwé et al., 2016). Guiding breeding programs requires also much more knowledge on stress physiology of a given species with focus on carbohydrate supply within a tree during different stress levels and the processes related to recovery from embolism at the cellular and whole plant level (Zwieniecki and Secchi, 2015).

## Conclusions and outlook

Regarding the aims of our study, (i) to develop proxies for *P*_50_ based on anatomy and (ii) to test these proxies for their plasticity regarding climate, in order to (iii) interpret annual variations of hydraulic proxies related to top dieback, we arrived at the following conclusions.

- The high predictive quality of (*t*/*b*)^2^ based on tangential lumen diameters for *P*_50_ implies, that, if we solely focus on radial cell dimensions as basis for this proxy we will lose a lot of information regarding hydraulic vulnerability of Norway spruce trunkwood. Calculating a mean diameter (e.g., derived from the lumen area), is preferable to the radial diameter, since information of the tangential diameter is included. The next step is to test these relationships within a given species, thus between different organs of a tree, and on other conifer species.
- Proxies for vulnerability to cavitation (*P*_50_) are influenced by climate. Traits associated with *P*_50_ were strongest related to precipitation in September of the previous growing season (high precipitation results in more vulnerable annual rings in the next season) but not to temperature. Warm but not too wet Septembers could result in higher carbon fixation with little impact on the hydraulic vulnerability of the annual ring formed in next season. High temperatures in April had a positive impact on all (*t*/*b*)^2^ traits. Norway spruce might be thus able to adapt to some extent to warmer spring temperatures that have been observed recently in this region. To our knowledge, this was the first attempt to analyze climate-hydraulic proxy relationships; similar studies shall be carried out for other conifer species.
- Investigating hydraulic vulnerability in a narrow time window may lead to misinterpretations on a plant's predisposition to drought stress under the impact of extreme climatic events, whereas chronologies of reliable vulnerability proxies (proofed by practical experiments!) are helpful to draw the whole picture. For instance, in 2010, trees prone to dieback were hydraulically less vulnerable than healthy looking trees indicated by their higher (*t*/*b*_ht_)^2^; this lower vulnerability developed however throughout a stress history starting from 2005. If one would investigate the year 2010 exclusively, the picture was as expected: anisohydry (less strict stomatal control) coupled with lower vulnerability to cavitation. Higher (*t*/*b*_ht_)^2^ was however the reaction to prolonged stress and not a general strategy to avoid drought stress. In fact, trees prone to top dieback produced less hydraulically safe wood under sufficient water supply (before 2005). This “opportunistic strategy” of producing more vulnerable wood when e.g., precipitation in the previous September and in May was high together with less strict stomatal control might have triggered top dieback. It remains to be tested if hydraulic survival strategies of individual trees of a given species change over time depending on their stress history.

## Author contributions

LD, SS had the project idea, were responsible for the project design, proposal writing and project management. JS, KA, IB, LD, and SR contributed to field work (site selection, tree selection, coring, harvesting, and sample pre-preparation), in that regard, site and tree selection was one of the biggest challenges. Hydraulic measurements were conducted by SR. SL was responsible for sample preparation for SilviScan measurements. RE assessed the SilviScan dataset and gave important input concerning data analyses. OT provided climate raw data and calculated daily water deficits. The dataset was analyzed by SR after pre-preparation steps done by JS, OT, and RE. All authors contributed to the interpretation of the results. SR wrote a first draft of the manuscript; thereafter all authors revised the first draft by rewriting, discussion and commenting. All authors were involved in re-writing the text and creating new graphs after the first revision of the reviewers. All authors agree on the contents of this manuscript.

## Funding

This study was mainly financed by the Norwegian Research Council (project “Dieback in Norway spruce”, No. 199403), by the Norwegian Forest Owners' Research Fund “Skogtiltaksfondet”, six regional funds in Norway (Fylkesmannen), by the Austrian Science Fund FWF (V146-B16) and by the CZ Ministry of Education (No. 6215648902). The research leading to these results has also received funding (employment of SL) from the European Union's Seventh Framework Programme for research, technological development and demonstration under grant agreement n° 284181 (“Trees4Future”). The contents of this publication reflect only the authors' views and the European Union is not liable for any use that may be made of the information contained therein.

### Conflict of interest statement

The authors declare that the research was conducted in the absence of any commercial or financial relationships that could be construed as a potential conflict of interest.

## Supplementary material

Supplementary Figure 1 **Mean monthly precipitation and daily water deficit from 1957 to 2015 of Sande and Hoxmark sites (gray areas or bars)**. Red lines indicate the year 1992, where early summer was extremely dry, blue lines indicate the extremely wet year 2007.

Supplementary Figure 2 **Mean monthly temperature (A), precipitation (B) and daily water deficit during the vegetation period (May–September) of years 1989–2010**. Bars represent mean values for Sande and Hoxmark.

Supplementary Table 1 **Information on the origin of the wood beam sample set for calculation of *P*_50_ and anatomical proxies from the SilviScan data set (*n* = 19) of 12 trees, where six trees were healthy looking and six trees showed signs of top dieback**.

## Abbreviations

DBH: diameter at breast height [cm]
*b*_h_: hydraulic lumen diameter [*μm*]
*b*_hr_: radial hydraulic lumen diameter [*μm*]
*b*_ht_: tangential hydraulic lumen diameter [*μm*]
*b*_r_: radial lumen diameter [*μm*]
*b*_t_: tangential lumen diameter [*μm*]
*d*_r_: radial tracheid diameter, from one middle lamellae to the next [*μm*]
*d*_t_: tangential tracheid diameter, from one middle lamellae to the next [*μm*]
*t*: double wall thickness [*μm*]
*P*_50_: negative of the overpressure inducing 50 % loss of hydraulic conductivity
RF: number of radial tracheid files/mm circumference
RW: ring width [mm]
SE: standard error
TF: number of tangential tracheid files/annual ring
(*t*/*b*_h_)^2^: conduit wall reinforcement of cells with ±10% *b*_h_
(*t*/*b*_hr_)^2^: conduit wall reinforcement of cells with ±10% *b*_hr_
(*t*/*b*_ht_)^2^: conduit wall reinforcement of cells with ±10% *b*_ht_.
