# Supplementary figures and images for: Novel Hydraulic Vulnerability Proxies for a Boreal Conifer Species Reveal That Opportunists May Have Lower Survival Prospects under Extreme Climatic Events
Source: Front Plant Sci. 2016 Jun 9;7:831. doi: 10.3389/fpls.2016.00831 (PMC4899478; doi:10.3389/fpls.2016.00831)

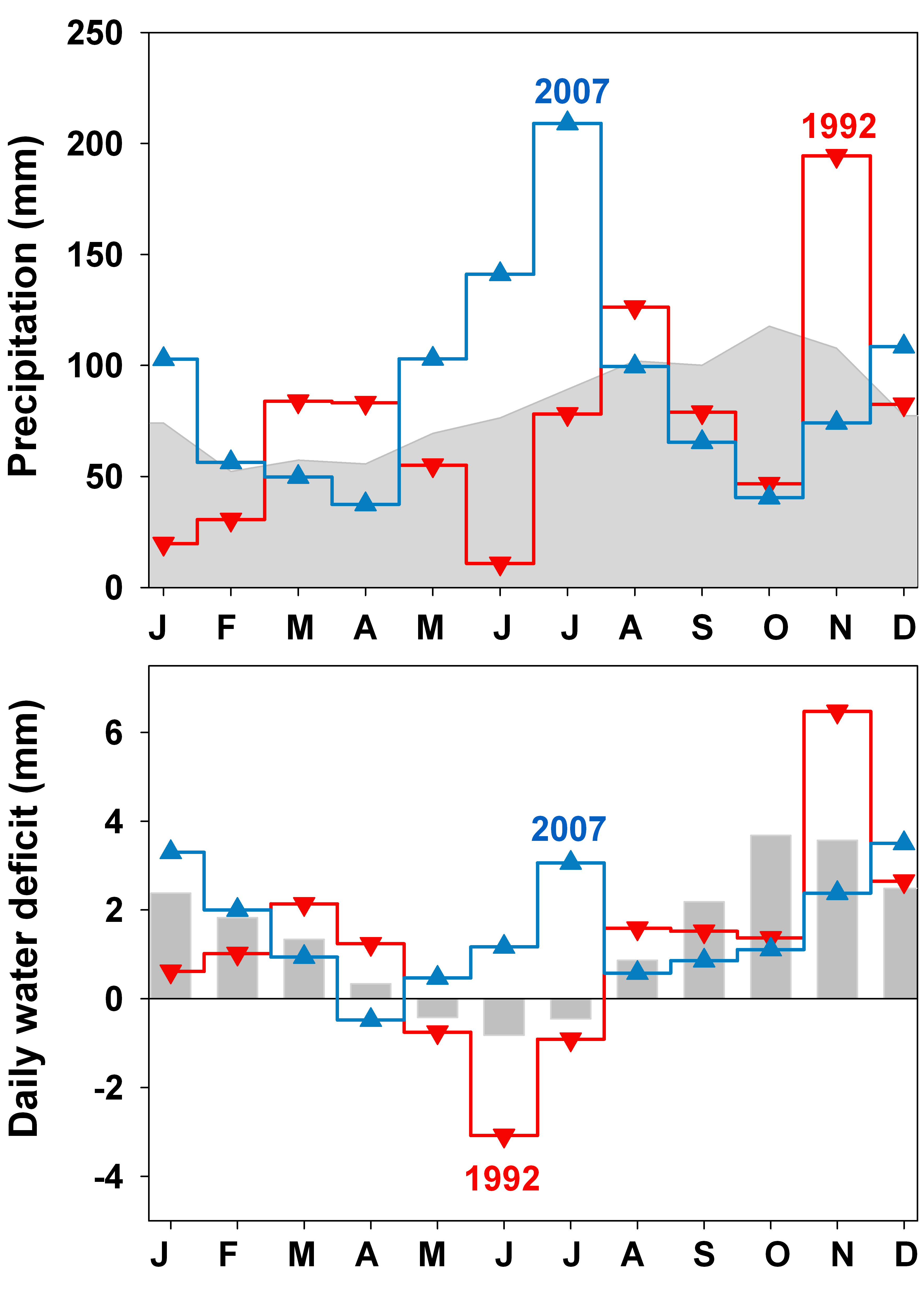

Supplement: Supplementary Figure 1 — Mean monthly precipitation and daily water deficit from 1957 to 2015 of Sande and Hoxmark sites (gray areas or bars). Red lines indicate the year 1992, where early summer was extremely dry, blue lines indicate the extremely wet year 2007. [file Image1.JPEG]

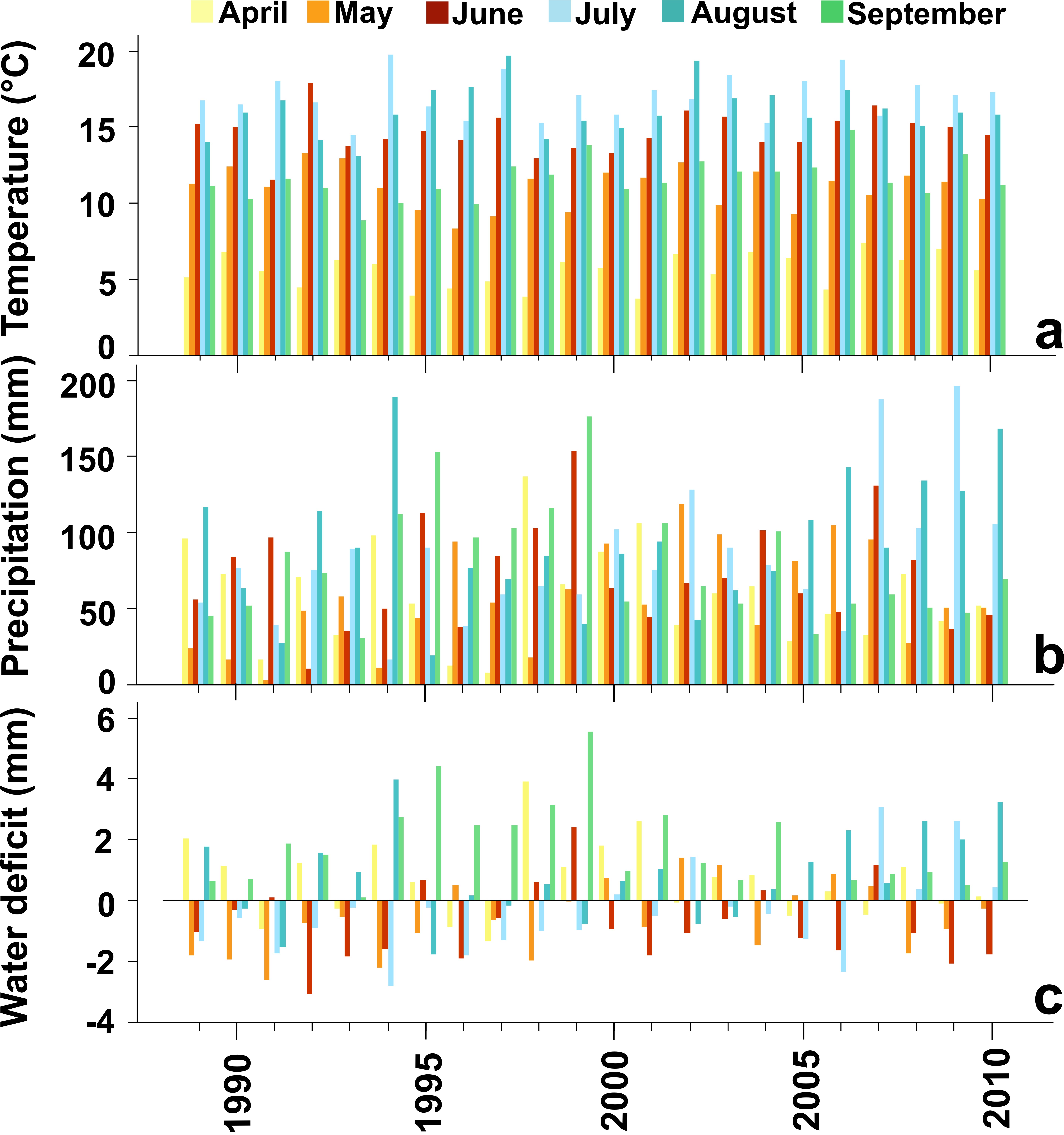

Supplement: Supplementary Figure 2 — Mean monthly temperature (A), precipitation (B) and daily water deficit during the vegetation period (May–September) of years 1989–2010. Bars represent mean values for Sande and Hoxmark. [file Image2.jpg]
