# Supplementary material for: Novel Hydraulic Vulnerability Proxies for a Boreal Conifer Species Reveal That Opportunists May Have Lower Survival Prospects under Extreme Climatic Events
Source: Front Plant Sci. 2016 Jun 9;7:831. doi: 10.3389/fpls.2016.00831 (PMC4899478; doi:10.3389/fpls.2016.00831)
Supplement: Supplementary Table 1 — Information on the origin of the wood beam sample set for calculation of P50 and anatomical proxies from the SilviScan data set (n = 19) of 12 trees, where six trees were healthy looking and six trees showed signs of top dieback. [file Table1.docx]

**Supplement Table**

**TABLE 1 Supplement** | Information on the origin of the wood beam sample set for calculation of *P*_50_ and anatomical proxies from the SilviScan data set (n = 19) of twelve trees, where six trees were healthy looking and six trees showed signs of top dieback.

| **Beam Nr.** | **Internal code** | **Site** | **Plot** | **Health state** | **Tree ID** |
| --- | --- | --- | --- | --- | --- |
| 1 | 205 | Sande | 1 | healthy looking | 1 |
| 2 | 208 | Sande | 1 | top dieback | 7 |
| 3 | 211 | Sande | 3 | healthy looking | 2 |
| 4 | 230 | Sande | 6 | top dieback | 8 |
| 5 | 237 | Sande | 7 | healthy looking | 3 |
| 6 | 238 | Sande | 7 | healthy looking | 3 |
| 7 | 240 | Sande | 7 | top dieback | 9 |
| 8 | 242 | Sande | 7 | top dieback | 9 |
| 9 | 243 | Sande | 7 | top dieback | 9 |
| 10 | 246 | Sande | 8 | healthy looking | 4 |
| 11 | 248 | Sande | 8 | healthy looking | 4 |
| 12 | 250 | Sande | 8 | healthy looking | 4 |
| 13 | 262 | Sande | 8 | top dieback | 10 |
| 14 | 266 | Sande | 9 | healthy looking | 5 |
| 15 | 268 | Sande | 9 | healthy looking | 5 |
| 16 | 270 | Sande | 9 | top dieback | 11 |
| 17 | 279 | Hoxmark | 2 | healthy looking | 6 |
| 18 | 280 | Hoxmark | 2 | healthy looking | 6 |
| 19 | 292 | Hoxmark | 4 | top dieback | 12 |
